# Insecticidal Suppression of Asian Citrus Psyllid *Diaphorina citri* (Hemiptera: Liviidae) Vector of Huanglongbing Pathogens

**DOI:** 10.1371/journal.pone.0112331

**Published:** 2014-12-01

**Authors:** Jawwad A. Qureshi, Barry C. Kostyk, Philip A. Stansly

**Affiliations:** University of Florida, Entomology and Nematology Department, Institute of Food and Agricultural Sciences, Southwest Florida Research and Education Center, Immokalee, Florida, United States of America; Kansas State University, United States of America

## Abstract

*Diaphorina citri* vectors pathogens that cause ‘huanglongbing’ or citrus greening disease which poses a serious threat to citrus production worldwide. Vector suppression is critical to reduce disease spread. Efficacy is a main concern when choosing an insecticide. Insecticidal treatments of 49 products or 44 active ingredients (a.i) labeled or experimental were field tested between 2005–2013 as foliar sprays (250 treatments, 39 a.i) or soil applications (47 treatments, 9 a.i) to control *D. citri* in citrus. A combined effect of nymphal and adult suppression in response to sprays of 23 insecticides representing 9 modes of action (MoA) groups and 3 unknown MoA provided more than 90% reduction of adult *D. citri* over 24–68 days. Observable effects on nymphs were generally of shorter duration due to rapid maturation of flush. However, reduction of 76–100% nymphs or adults over 99–296 days was seen on young trees receiving drenches of the neonicotinoids imidacloprid, thiamethoxam or clothianidin (MoA 4A) and a novel anthranilic diamide, cyantraniliprole (MoA 28). Effective products identified for foliar sprays to control *D. citri* provide sufficient MoA groups for rotation to delay evolution of insecticide resistance by *D. citri* and other pests. However, cyantraniliprole is now the only available alternative for rotation with neonicotinoids in soil application to young trees. Sprays of up to eight of the most effective insecticides could be rotated over a year without repetition of any MoA and little or no recourse to neonicotinoids or cyantraniliprole, so important for protection of young trees. Other considerations effecting decisions of what and when to spray include prevalence of huanglongbing, pest pressure, pre-harvest intervals, overall budget, equipment availability, and conservation of beneficial arthropods. Examples of spray programs utilizing broad-spectrum and relatively selective insecticides are provided to improve vector management and may vary depending on individual or regional assessment of all factors.

## Introduction

*Diaphorina citri* Kuwayama, also known as Asian citrus psyllid (ACP), is a global pest of citrus and vector of “*Candidatus* Liberibacter” pathogens responsible for causing ‘huanglongbing’ (HLB) or citrus greening disease [1], [2], [3]. Huanglongbing is one of the world's most devastating diseases of citrus, responsible for tree decline and loss of production in disease affected regions [4], [5]. In the United States, *D. citri* was first discovered in Palm Beach County, Florida on hedges of orange jasmine, *Murraya paniculata* (L.) Jack. (Rutaceae) in 1998 [6] and quickly established in citrus producing regions of the state [7], [8], [9]. First detection in the USA of the Asian form of HLB occurred in south Miami Dade during August 2005 [10]. The disease now occurs throughout the state and threatens a citrus industry which generates $9 billion in annual revenues [11], [12]. A recent study estimated that due to reduced citrus production in Florida, total cumulative production and revenue were reduced by 23% and 16%, respectively, and 48% of total jobs lost in the Agriculture, Forestry and Fisheries sector, over the five-year period since 2006 [13]. HLB was identified from Louisiana in 2008 and from South Carolina and Georgia in 2009 (http://www.aphis.usda.gov/plant_health/plant_pest_info/) and is also present in Texas, Mississippi and California [14], [15].

New foliage growth (flush) regulates the dynamics of several citrus pests requiring soft tissues for oviposition and development including *D. citri*, citrus leafminer, *Phyllocnistis citrella* Stainton (Lepidoptera: Gracillariidae) and several aphid species. Flush production is influenced by weather, scion and rootstock variety and plant age [16]. Young trees flush frequently compared to mature trees and therefore need more protection from these pests. The typical pattern of shoot production in mature citrus trees in Florida begins with a major flush in late winter or early spring, a lesser flush in the early summer and minor flushes during late summer and fall, followed by a relatively dormant winter season in late fall and early winter with little or no new foliage growth [9], [17].

Reproduction of *D. citri* is totally dependent on availability of young shoots containing feather stage to recently expanded tender leaves. Female psyllids must feed on tender shoots to mature eggs and prefer opening buds and emerging shoots for oviposition. During the following 2–3 weeks, shoot and leaf tissues are still tender and are utilized by nymphs and adults respectively to complete development and mature eggs. Adults can also feed and survive on the fully developed leaves for several months [8], [18].

Complete control of HLB may not be feasible until plants expressing high levels of resistance to the vector and/or disease are available. Meanwhile, an integrated strategy involving biological and chemical control tactics is required for sustainable management of the pest to reduce disease spread. Biological control has always been an important component of citrus insect pest management in Florida [19] including *D. citri*. Lady beetles, lacewings and spiders are all well-known as predators of citrus psyllids [18], [20], [21], [22], [23]. These and other predators were observed to inflict 80–100% mortality to *D. citri* immature and were abundant during spring and summer, though largely absent during winter in concert with citrus growth patterns and psyllid abundance [21], [22]. Two exotic Hymenopteran parasitoids of ACP, *Diaphorencyrtis aligarhensis* (Shafee, Alam and Agaral) (Encyrtidae) and *Tamarixia radiata* (Waterston) (Eulophidae) were introduced in Florida in 2000 [24]. *Tamarixia radiata* is now widely distributed in the Florida citrus ecosystem at variable rates of parasitism and new strains are being released to enhance parasitism rates, whereas *D. aligarhensis* has not yet established [9], [25]. Growers in Florida have also released the convergent lady beetle *Hippodamia convergens* Guérin-Méneville from California; however, it is still not common there in citrus [26]. Although combined effects of natural enemies have not proven sufficient to prevent spread of HLB, nevertheless, conservation of these and other beneficial arthropods is essential for effective citrus pest management in Florida and elsewhere.

Insecticides are presently a critical component of ACP management. The systemic neonicotinoid insecticides, thiamethoxam, imidacloprid and clothianidin and a new insecticide cyantraniliprole are allowed in Florida citrus but their use as soil applications is limited by rate restrictions to young trees [27]. Aldicarb (Temik 15 G) was used on large trees in Florida but is no longer permitted [18]. Suppression of ACP by sprays of broad-spectrum insecticides prior to flushing has proved to be an effective strategy for reducing populations. This is especially true of sprays during tree dormancy to target the overwintering adult population [23], [28]. Nevertheless, it is also necessary to control ACP during the growing season which in Florida commences with spring flush and lasts through mid-fall.

The advent of HLB has greatly intensified insecticide use to control ACP in Florida citrus [29], [30]. However, timing, choice of products, and application methods during the growing season are far from standard. Factors such as overall budget, efficacy, pest pressure, equipment availability, conservation of beneficial insects and resistance management all warrant consideration. We have extensively field tested recommended and experimental insecticides in replicated experiments against ACP since 2005. This manuscript reports data on the effectiveness of the tested insecticides based on the number of days significantly fewer ACP were observed on the treated trees compared to the untreated control trees and degree of ACP reduction. The intent is to facilitate management decisions to treat citrus groves for ACP individually or on an area wide basis (www.flchma.org).

## Materials and Methods

### Study location and experimental trees

Experiments were conducted at the Southwest Florida Research and Education Center, of the University of Florida-IFAS, Immokalee, FL, USA (Latitude: 26.484 N, Longitude: 81.435 W) and a neighboring commercial grove located near Labelle, FL, USA (Latitude: 26.693 N, Longitude: 81.446 W). No permit or specific permission was required. These studies did not involve endangered or protected species. Most tests for effects of foliar sprays on ACP were made on ‘Valencia’ sweet orange *Citrus sinensis* (L.) Osbeck (Rutaceae) trees planted in 1998 at a density of 326 trees/ha on double-row raised beds. Trees were pruned with hand held or tractor mounted hedger to encourage growth of new shoots to support ACP infestation. Soil applied insecticides were tested in 2–5 yr old orange trees flushing naturally. Trees were irrigated by micro sprinklers and subjected to conventional cultural practices [31]. There was little or no use of insecticides at the study locations. Psyllids in these experiments were feral and originated at locations where studies were conducted. Surrounding conventional groves employed chemical control to suppress psyllids.

### Experimental design

All experiments were designed as randomized complete blocks (RCB) with four replicates of each treatment. Four to 15 trees in a single row were used for each replicate of a treatment. Replicates within treated rows were separated from each other by one untreated tree and sprayed rows were separated from each other by an untreated buffer row to avoid spray drift between plots within and between treated rows. Testing was done during the growing season and young shoots required by psyllids to develop and reproduce were available either naturally or induced through pruning.

### Treatment application

Two hundred and ninety seven insecticidal treatments of 49 products or 44 active ingredients (a.i) (Table 1) representing 11 insecticide mode of action (MoA) (http://www.irac-online.org) groups and 8 unknown MoA were field tested as foliar sprays (250 treatments, 39 a.i) (Tables 2–4) and soil applications (47 treatments, 9 a.i) (Table 5) against ACP in citrus between 2005–2013. Rates tested were within the range recommended for use or being investigated for experimental use.

**Table 1:** Name, active ingredient, mode of action group and manufacturer of the products tested as foliar spray or soil application against *Diaphorina citri*.

Product^a^	Active ingredient (% by weight)	IRAC MOA Group^b^	Manufacturer
435 oil	horticultural mineral oil (98.8)	NA	Drexel Chemical Company, Memphis, TN
Actara 25 WG	thiamethoxam (25)	4A	Syngenta Crop Protection, LLC, Greensboro, NC
Admire Pro 4.6 SC	imidacloprid^c^ (42.8)	4A	Bayer CropScience LP, Research Triangle Park, NC
Agri-Flex	abamectin + thiamethoxam (3+13.9)	6+4A	Syngenta Crop Protection, LLC, Greensboro, NC
Agri-Mek 0.15 EC	abamectin^c^ (2)	6	Syngenta Crop Protection, LLC, Greensboro, NC
Apta 15 SC	tolfenpyrad (15)	21A	Nichino America Inc., Wilmington, DE
Assail 30 SG	acetamiprid (30)	4A	Cerexagri, Inc., King of Prussia, PA
Aza-Direct	azadirachtin (1.2)	NA	Gowan Company, Yuma, AZ
Belay 2.13 SC	clothianidin (23)	4A	Valent USA Corporation, Walnut Creek, CA
Beleaf 50 SG	flonicamid (50)	9C	FMC Corporation, Philadelphia, PA
Belt 4 SC	flubendiamide (39)	28	Bayer CropScience LP, Research Triangle Park, NC
Baythroid XL	beta-cyfluthrin (12.7)	3A	Bayer CropScience LP, Research Triangle Park, NC
Closer SC	sulfoxaflor (21.8)	4C	Dow AgroSciences, LLC, Indianapolis, IN
Danitol 2.4 EC	fenpropathrin (30.9)	3A	Valent USA Corporation, Walnut Creek, CA
Delegate WG	spinetoram (25)	5	Dow AgroSciences, LLC, Indianapolis, IN
Dibrom 8 E	naled (62)	1B	Amvac Chemical Corporation, Los Angeles, CA
Dimethoate 4 E	dimethoate (43.5)	1B	Cheminova, Inc., Wayne, NJ
Exirel	cyantraniliprole (10.2)	28	DuPont Company, Newark, DE
Entrust SC	spinosad (22.5)	5	Dow AgroSciences, LLC, Indianapolis, IN
Envidor 2 SC	spirodiclofen (22.3)	23	Bayer CropScience LP, Research Triangle Park, NC
Fulfill 50 WDG	pymetrozine (50)	9B	Syngenta Crop Protection, LLC, Greensboro, NC
Grandevo	*Chromobacterium subtsugae* ^c^ (30)	NA	Marrone Bio Innovations, Davis, CA
Imidan 70 W	phosmet (70)	1B	Gowan Company, Yuma, AZ
Lorsban 4 E	chlorpyrifos^c^ (44.9)	1B	Dow AgroSciences, LLC, Indianapolis, IN
Magus	fenazaquin (18.8)	21A	Gowan Company, Yuma, AZ
Micromite 80 WGS	diflubenzuron (80)	15	Chemtura Corporation., Middlebury, CT
Movento 240 SC	spirotetramat^c^ (22.4)	23	Bayer CropScience LP, Research Triangle Park, NC
MBI-206 EP	*Burkholderia spp* (NA)	NA	Marrone Bio Innovations, Davis, CA
M-Pede	potassium salts of fatty acids (49)	NA	Dow Agrosciences, LLC, Indianapolis, IN
MSR 2 E	oxydemeton-methyl (25)	1B	Gowan Company, Yuma, AZ
Mustang Max 1.5 EC	zeta-cypermethrin^c^ (9.6)	3A	FMC Corporation, Philadelphia, PA
Nexter	pyridaben (75)	21A	Gowan Company, Yuma, AZ
NNI0101-20SC	pyrifluquinazon (NA)	NA	Nichino America Inc., Wilmington, DE
NoFly WP	*Isaria fumosoroseus* strain FE 9901(18)	NA	Natural Industries Inc., Spring, TX
NUQ 05054	imidacloprid (NA)	4A	Nufarm Americas Inc., Burr Ridge, IL
Platinum 75 SG	thiamethoxam^c^ (75)	4A	Syngenta Crop Protection, Greensboro, NC
Portal	fenpyroximate (5)	21A	Nichino America Inc., Wilmington, DE
Requiem 25 EC	synthetic extract of *Chenopodium ambrosioides* (25)	NA	AgraQuest, Davis, CA
Sevin XLR	carbaryl (42.8)	1A	Bayer CropScience LP, Research Triangle Park, NC
Sil-Matrix	potassium silicate (29)	NA	PQ Corporation, Valley Forge, PA
Sivanto 200 SL	flupyradifurone (NA)	4D	Bayer CropScience LP, Research Triangle Park, NC
Stallion	chlorpyrifos + zeta-cypermethrin (30.8+3.08)	1B+3A	FMC Corporation, Philadelphia, PA
Supracide 2 E	methidathion (24.4)	1B	Gowan Company, Yuma, AZ
Temik 15 G	aldicarb (15)	1A	Bayer CropScience LP, Research Triangle Park, NC
Venom 70 SG	dinotefuran (70)	4A	Valent Biosciences Corporation, Libertyville, IL
Verimark	cyantraniliprole (18.7)	28	DuPont Company, Newark, DE
VoliamFlexi	chlorantraniliprole + thiamethoxam (20+20)	28+4A	Syngenta Crop Protection, Greensboro, NC
Vydate L	oxamyl (24)	1A	DuPont Company, Newark, DE
Warrior II	lambda-cyhalothrin (22.8)	3A	Syngenta Crop Protection, Greensboro, NC

a Not all products are permitted for use on orange, so always follow the label for details on proper use.

b Insecticide Resistance Action Committee (IRAC) Mode of Action (MOA) Scheme, Version 7.3, April 2014. ^c^More than one formulation or product brand tested. NA  =  Not available

**Table 2:** Duration and magnitude of reduction of *Diaphorina citri* adults on orange trees treated with foliar sprays of insecticides in Florida.

Product^a^	Active ingredient		Rates tested		Duration of adult reduction					Magnitude of reduction				
			(oz/acre)			(days)^d^					(%)^d^			
		N^c^	Low	High	Low	High	Mean	± SEM	Rank^e^	Low	High	Mean	± SEM	Reference^f^
Exirel	cyantraniliprole	1(1)	16	16	60	60	60	0	1	80	80	80	0	[56]
Apta 15 SC	tolfenpyrad	8(8)	11	24	17	68	57	6	2	77	99	90	3	[34], [35], [55]
VoliamFlexi	chlorantraniliprole + thiamethoxam	4(3)	5	7.5	24	67	46	12	3	82	100	90	4	[45], [56], [60]
Sivanto 200 SL	flupyradifurone	18(10)	6.8	14	19	52	37	3	4	47	90	73	3	[37], [38], [56], [61], [62]
Actara 25 WG	thiamethoxam	10(4)	2.8	5.5	24	60	36	4	5	58	100	84	5	[39], [43], [44], [45], [56]
Warrior II	lamda-cyhalothrin	3(0)	1.4	5.8	31	42	35	4	6	88	97	92	3	[39], [40]
Baythroid XL	beta-cyfluthrin	3(0)	3	3	18	51	35	10	7	62	87	79	9	[41], [42], [62]
Supracide 2 E	methidathion	1(0)	32	32	33	33	33	0	8	91	91	91	0	[46]
Danitol 2.4 EC	fenpropathrin	8(5)	16	21.3	7	60	33	6	9	68	100	90	4	[38], [39], [40], [45], [46], [47], [48], [56]
Portal	fenpyroximate	10(7)	24	64	17	58	33	4	10	59	97	76	5	[34], [43], [45], [49], [55], [57], [61]
Dibrom 8 E	naled	2(1)	16	16	24	42	33	9	11	52	91	71	19	[45], [57]
Closer SC	sulfoxaflor	16(15)	2.9	5.7	12	67	32	4	12	17	93	62	7	[38], [56], [59], [60]
Delegate WG	spinetoram	21(19)	4	6	16	52	32	2	13	66	98	89	2	[37], [38], [43], [48], [49], [50], [58], [59], [63]
Agri-Flex	abamectin + thiamethoxam	5(5)	8.5	8.5	17	53	31	6	14	82	100	92	4	[34], [44], [45], [56], [60]
Dimethoate 4 E	dimethoate	1(0)	16	16	31	31	31	0	15	96	96	96	0	[40]
Agri-Mek 0.15 EC	abamectin^b^	4(4)	4.3	20	24	42	31	4	16	32	87	54	13	[39], [43], [56]
Mustang Max 1.5 EC	zeta-cypermethrin^b^	5(0)	4	4.3	18	44	30	5	17	44	97	75	9	[41], [42], [48], [62]
Lorsban 4 E	chlorpyrifos^b^	8(4)	24	80	24	42	29	3	18	79	100	91	4	[40], [48], [52], [57]
Magus	fenazaquin	7(7)	16	36	26	42	28	4	19	58	98	66	11	[46], [57], [61]
Movento 240 SC	spirotetramat	24(22)	5	16	0	58	26	4	20	0	97	60	6	[34], [36], [37], [38], [41], [42], [44], [49], [51], [62]
Stallion	chlorpyrifos + zeta-cypermethrin	1(1)	11.8	11.8	24	24	24	0	21	100	100	100	0	[52]
Sil-Matrix	potassium silicate	1(0)	128	128	24	24	24	0	22	74	74	74	0	[50]
Admire Pro 4.6 SC	imidacloprid^b^	9(5)	1	7	0	67	22	8	23	0	88	60	9	[36], [37], [43], [51], [53], [56]
Micromite 80 WGS	diflubenzuron	10(9)	3.1	6.3	0	48	20	6	24	0	94	46	9	[34], [36], [39], [43], [49], [54]
Grandevo	*Chromobacterium subtsugae* b	11(10)	24	256	0	28	20	2	25	0	97	64	8	[45], [58], [63]
Imidan 70 W	phosmet	7(0)	8	24	0	33	18	6	26	0	100	51	18	[40], [46], [52]
MBI-206 EP	*Burkholderia spp*	5(5)	192	384	7	28	18	4	27	48	99	69	9	[45], [58], [63]
435 oil	horticultural mineral oil	11(0)	192	640	0	38	18	4	28	0	76	36	10	[34], [36], [37], [43], [44], [49], [50], [52], [54], [58], [63]
Belt 4 SC	flubendiamide	2(2)	5	7.5	17	17	17	0	29	36	52	44	6	[34]
Fulfill 50 WDG	pymetrozine	1(1)	5.5	5.5	17	17	17	0	30	38	38	38	0	[60]
Entrust SC	spinosad	1(1)	3	3	16	16	16	0	31	86	86	86	0	[63]
MSR 2 E	oxydemeton-methyl	3(0)	24	48	0	24	16	6	32	0	59	33	18	[36], [43]
Sevin XLR	carbaryl	2(0)	48	48	14	17	16	2	33	4	74	39	35	[51], [54]
NoFly WP	*Isaria fumosoroseus*	2(0)	16	32	16	16	16	0	34	48	53	51	3	[63]
Requiem 25 EC	*Chenopodium ambrosioides*	12(5)	64	192	0	24	13	3	35	0	76	47	9	[43], [47], [49], [50], [54]
M-pede	potassium salts of fatty acids	2(2)	106	393	0	24	12	12	36	0	97	49	49	[34], [45]
Nexter	pyridaben	5(1)	6.6	10.6	0	24	9	5	37	0	100	43	19	[36], [52], [57]
Vydate L	oxamyl	2(0)	32	64	7	7	7	0	38	71	79	75	4	[47]
Assail 30 SG	acetamiprid	1(0)	7	7	0	0	0	0	39	0	0	0	0	[53]
Aza-Direct	azadirachtin	1(0)	8	8	0	0	0	0	40	0	0	0	0	[46]
Envidor 2 SC	spirodiclofen	1(0)	20	20	0	0	0	0	41	0	0	0	0	[36]
NNI-0101	pyrifluquinazon	1(0)	6.4	6.4	0	0	0	0	42	0	0	0	0	[35]

a Not all products are permitted for use on orange, so always follow the label for details on proper use.

b More than one formulation or product brand tested.

c Number of times product was tested (in parenthesis with adjuvants) in a randomized complete block design using at least 20 trees in four replicates each time.

d Significantly more reduction compared to untreated control (P<0.05).

e Based on mean number of days significantly fewer adults were observed on treated trees compared to untreated trees.

f Reference to reports which appeared in non-peer reviewed literature.

**Table 3:** Duration and magnitude of reduction of *Diaphorina citri* nymphs on orange trees treated with foliar sprays of insecticides in Florida.

Product^a^	Active ingredient		Rates tested		Duration of nymphal reduction					Magnitude of reduction				
			(oz/acre)			(days)^d^					(%)^d^			
		N^c^	Low	High	Low	High	Mean	± SEM	Rank^e^	Low	High	Mean	± SEM	Reference^f^
Baythroid XL	beta-cyfluthrin	3(0)	3	3	15	51	30	11	1	63	84	73	6	[41], [42], [62]
Imidan 70 W	phosmet	7(0)	8	24	17	38	27	3	2	73	100	87	3	[40], [46], [52]
Lorsban 4 E	chlorpyrifos^b^	8(4)	24	80	17	38	27	3	3	74	98	84	3	[40], [48], [52], [57]
Exirel	cyantraniliprole	1(1)	16	16	24	24	24	0	4	75	75	75	0	[56]
Apta 15 SC	tolfenpyrad	8(8)	11	24	17	30	24	2	5	75	98	92	3	[34], [35], [55]
Closer SC	sulfoxaflor	16(15)	2.9	5.7	6	33	24	2	6	48	95	78	3	[38], [56], [59], [60]
Stallion	chlorpyrifos + zeta-cypermethrin	1(1)	11.8	11.8	24	24	24	0	7	72	72	72	0	[52]
Mustang Max 1.5 EC	zeta-cypermethrin^b^	5(0)	4	4.3	15	51	23	7	8	17	99	70	14	[41], [42], [48], [62]
Dibrom 8 E	naled	2(1)	16	16	21	24	23	2	9	62	87	74	13	[45], [57]
Movento 240 SC	spirotetramat	24(22)	5	16	0	51	23	3	10	59	99	82	3	[34], [36], [37], [38], [41], [42], [44], [49], [51], [62]
VoliamFlexi	chlorantraniliprole + thiamethoxam	4(3)	5	7.5	17	24	22	2	11	91	99	97	2	[45], [56], [60]
Agri-Flex	abamectin + thiamethoxam	5(5)	8.5	8.5	17	24	22	1	12	85	99	94	3	[34], [44], [45], [56], [60]
Danitol 2.4 EC	fenpropathrin	8(5)	16	21.3	7	42	22	4	13	78	93	86	2	[38], [39], [40], [45], [46], [47], [48], [56]
Magus	fenazaquin	7(7)	16	36	12	28	19	3	14	63	97	70	11	[46], [57], [61]
Sivanto 200 SL	flupyradifurone	18(10)	6.8	14	10	33	19	2	15	61	99	87	3	[36], [37], [38], [56], [61], [62]
Actara 25 WG	thiamethoxam	10(4)	2.8	5.5	3	24	18	3	16	61	99	89	4	[39], [43], [44], [45], [56]
Warrior II	lamda-cyhalothrin	3(0)	1.4	5.8	17	20	18	1	17	91	99	96	3	[39], [40]
Delegate WG	spinetoram	21(19)	4	6	3	28	18	2	18	78	98	92	1	[37], [38], [43], [48], [49], [50], [58], [59], [63]
Nexter	pyridaben	5(1)	6.6	10.6	0	24	18	5	19	0	62	31	13	[36], [52], [57]
Dimethoate 4 E	dimethoate	1(0)	8	8	17	17	17	0	20	96	96	96	0	[40]
Grandevo	*Chromobacterium subtsugae* b	11(10)	24	256	0	24	16	3	21	0	92	58	8	[45], [58], [63]
Portal	fenpyroximate	10(7)	24	64	0	24	15	3	22	0	98	66	7	[34], [43], [45], [49], [55], [57], [61]
MBI-206 EP	*Burkholderia spp*	5(5)	192	384	7	24	14	3	23	56	89	72	7	[45], [58], [63]
Belt 4 SC	flubendiamide	2(2)	5	7.5	10	17	14	3	24	46	67	57	8	[34]
Entrust SC	spinosad	1(1)	3	3	13	13	13	0	25	63	63	63	0	[63]
Supracide 2 E	methidathion	1(0)	32	32	12	12	12	0	26	77	77	77	0	[46]
Fulfill 50 WDG	pymetrozine	1(1)	5.5	5.5	12	12	12	0	27	66	66	66	0	[60]
M-pede	potassium salts of fatty acids	2(2)	106	393	0	24	12	12	28	0	90	45	45	[34], [45]
Agri-Mek 0.15 EC	abamectin^b^	4(4)	4.3	20	0	20	10	5	29	0	84	59	20	[39], [43], [56]
Sil-Matrix	potassium silicate	1(0)	128	128	10	10	10	0	30	67	67	67	0	[50]
Admire Pro 4.6 SC	imidacloprid^b^	9(5)	1	7	3	17	10	2	31	44	94	73	6	[36], [37], [43], [51], [53], [56]
Micromite 80 WGS	diflubenzuron	10(9)	3.1	6.3	0	27	10	4	32	0	87	44	12	[34], [36], [39], [43], [49], [54]
435 oil	horticultural mineral oil	11(0)	192	640	0	24	9	3	33	0	78	50	9	[34], [36], [37], [43], [44], [49], [50], [52], [54], [58], [63]
Vydate L	oxamyl	2(0)	32	64	7	7	7	0	34	59	72	65	6	[47]
Requiem 25 EC	*Chenopodium ambrosioides*	12(5)	64	192	0	17	6	2	35	0	78	35	9	[43], [47], [49], [50], [54]
MSR 2 E	oxydemeton-methyl	3(0)	24	48	3	10	5	2	36	53	96	69	14	[36], [43]
NoFly WP	*Isaria fumosoroseus*	2(0)	16	32	3	7	5	2	37	0	40	20	20	[63]
Assail 30 SG	acetamiprid	1(0)	7	7	3	3	3	0	38	40	40	40	0	[53]
Envidor 2 SC	spirodiclofen	1(0)	20	20	3	3	3	0	39	43	43	43	0	[36]
Sevin XLR	carbaryl	2(0)	48	48	0	3	2	2	40	0	90	45	45	[51], [54]
Aza-Direct	azadirachtin	1(0)	8	8	0	0	0	0	41	0	0	0	0	[46]
NNI-0101	pyrifluquinazon	1(0)	6.4	6.4	0	0	0	0	42	0	0	0	0	[35]

a Not all products are permitted for use on orange, so always follow the label for details on proper use.

b More than one formulation or product brand tested.

c Number of times product was tested (in parenthesis with adjuvants) in a randomized complete block design using at least 20 trees in four replicates each time.

d Significantly more reduction compared to untreated control (P<0.05).

e Based on mean number of days significantly fewer nymphs were observed on treated trees compared to untreated trees.

f Reference to reports which appeared in non-peer reviewed literature.

**Table 4:** Duration and magnitude of reduction of *Diaphorina citri* adults and nymphs on orange trees sprayed with same rate of an insecticide alone or with horticultural mineral oil at 2-3% of application volume.

Product^a^	Active ingredient	Product rate	Duration of significant reduction (days)^b^			Magnitude of reduction (%)^b^		Reference^c^
		(oz/acre)	With adjuvant	Without adjuvant		With adjuvant	Without adjuvant	
			**ACP Adult**					
Sivanto 200 SL	flupyradifurone	7	21	21	58±16		60±20	[61]
Actara 25 WG	thiamethoxam	5.5	36	36	96±08		84±05	[44]
Danitol 2.4 EC	fenpropathrin	16	40	31	92±03		83±10	[38], [40]
Portal	fenpyroximate	64	24	24	61±09		59±12	[43]
Dibrom 8 E	naled	16	24	42	91±16		52±12	[45], [57]
Closer SC	sulfoxaflor	2.9	19	12	88±08		74±14	[38]
Delegate WG	spinetoram	4	24	17	96±02		98±02	[48]
Lorsban 4 E	chlorpyrifos	40	24	24	79±00		99±01	[48], [52]
Micromite 80 WGS	diflubenzuron	6.3	17	3	94±03		47±00	[34], [43]
Requiem 25 EC	*Chenopodium ambrosioides*	96	24	24	76±06		63±17	[50]
			**ACP Nymph**					
Sivanto 200 SL	flupyradifurone	7	21	14	80±05		92±03	[61]
Actara 25 WG	thiamethoxam	5.5	22	22	97±07		90±02	[44]
Danitol 2.4 EC	fenpropathrin	16	33	17	88±04		88±05	[38], [40]
Portal	fenpyroximate	64	10	10	43±08		48±20	[43]
Dibrom 8 E	naled	16	24	21	87±08		62±14	[45], [57]
Closer SC	sulfoxaflor	2.9	33	33	83±10		71±12	[38]
Delegate WG	spinetoram	4	10	10	100±00		96±01	[48]
Lorsban 4 E	chlorpyrifos	40	24	17	74±11		98±02	[48], [52]
Micromite 80 WGS	diflubenzuron	6.3	17	0	78±14		0	[34], [43]
Requiem 25 EC	*Chenopodium ambrosioides*	96	17	10	67±07		66±08	[50]

a Not all products are permitted for use on orange, so always follow the label for details on proper use including adjuvants.

b Significantly more reduction compared to untreated control (P<0.05). Duration of reduction is one number representing days significantly fewer *D. citri* were observed on treated trees compared to untreated trees. Magnitude of reduction is mean ± SEM calculated from reduction observed on multiple dates until significant effect was observed.

c Reference to reports which appeared in non-peer reviewed literature.

**Table 5:** Duration and magnitude of reduction of *Diaphorina citri* adults and nymphs on orange trees treated with soil applications of insecticides in Florida.

Product^a^	Active ingredient		Rates tested (oz/acre)		Duration of reduction (days)^f^					Magnitude of reduction (%)^f^				
		N^e^	Low	High	Low	High	Mean	± SEM	Rank^g^	Low	High	Mean	±SEM	Reference^h^
							**ACP adult**							
NUQ 05054^b^	imidacloprid	2	160	160	176	176	176	0	1	71	71	71	0	[66], [67]
Verimark^c^	cyantraniliprole	7	10.3	30.4	140	140	140	0	2	51	76	60	8	[64], [65], [68]
Belay 2.13 SC^c^	clothianidin	4	6	12	44	140	111	23	3	68	85	78	4	[68], [75]
Temik 15 G^b^	aldicarb	3	125	528	92	121	106	8	4	68	81	75	4	[66], [73]
Admire Pro 4.6 SC^c^	imidacloprid	12	4.7	16	37	153	84	14	5	50	94	79	5	[64], [65], [66], [67], [68], [69], [70], [71], [72], [74], [75]
Platinum 75 SG^c^	thiamethoxam^d^	7	2.7	18.8	43	99	81	13	6	65	95	85	7	[64], [65], [69], [70], [71]
Venom 70 SG^c^	dinotefuran	2	3	3.8	37	55	46	9	7	28	56	42	14	[69], [75]
Sivanto 200 SL^c^	flupyradifurone	6	14	28	0	62	43	9	8	0	81	47	12	[72], [74]
Movento MPC^c^	spirotetramat	2	8	16	0	42	21	21	9	0	78	39	39	[74]
Beleaf 50 SG^c^	flonicamid	3	6.3	20.8	0	37	20	11	10	0	46	18	14	[75]
							**ACP nymph**							
Verimark^c^	cyantraniliprole	7	10.3	30.4	70	296	185	38	1	73	100	84	4	[64], [65], [68]
NUQ 05054^b^	imidacloprid	2	160	160	142	223	183	41	2	42	45	44	2	[66], [67]
Admire Pro 4.6 SC^c^	imidacloprid	12	4.7	16	44	245	107	18	3	40	100	71	6	[64], [65], [66], [67], [68], [69], [70], [71], [72], [74], [75]
Platinum 75 SG^c^	thiamethoxam^d^	7	2.7	18.8	43	274	103	29	4	60	100	85	6	[64], [65], [69], [70], [71]
Belay 2.13 SC^c^	clothianidin	4	6	12	44	141	85	20	5	81	93	89	3	[68], [75]
Temik 15 G^b^	aldicarb	3	125	528	23	121	72	49	6	17	55	36	19	[66], [73]
Venom 70 SG^c^	dinotefuran	2	3	3.8	30	84	57	27	7	24	51	38	14	[69], [75]
Sivanto 200 SL^c^	flupyradifurone	6	14	28	0	55	32	8	8	0	65	41	11	[72], [74]
Beleaf 50 SG^c^	flonicamid	3	6.3	20.8	0	37	25	12	9	0	28	17	9	[75]
Movento MPC^c^	spirotetramat	2	8	16	0	0	0	0	10	0	0	0	0	[74]

a Not all products are permitted for use on orange, so always follow the label for details on proper use.

b Granular formulations, aldicarb applied within two, 3 ft furrows approximately 2 ft from the base of the two opposing sides of tree. NUQ 05054 is a slow release imidacloprid applied in a 4 ft circle around the base of the tree.

c Liquid formulations applied as drenches to bare soil at a radius of 24 inches around the trunk of the tree with an EZ-Dose sprayer at a pressure of 45 psi and a flow rate of 3.7 gallon per minute.

d More than one formulation tested.

e Number of times product was tested in a randomized complete block design using at least 20 trees in four replicates each time.

f Significantly more reduction compared to untreated control (P<0.05).

g Based on mean number of days significantly fewer adults or nymphs were observed on treated trees compared to untreated trees.

h Reference to reports which appeared in non-peer reviewed literature.

Sprays were applied using a Durand Wayland 3P-10C-32 or John Bean 400 Redline air blast speed sprayer delivering between 935 and 1402 L/ha (100-150 gallon/acre) final application volume depending upon the requirement of the product. Certain low volume treatments were applied with a Proptec rotary atomizer sprayer which delivered between 47-94 L/ha (5–10 gallon/acre). Some insecticides were evaluated with an adjuvant, mostly horticultural mineral oil (HMO). Without adjuvant comparisons are presented for insecticides evaluated at the same rate (Table 4).

Soil drench application of systemic insecticides were made to bare soil at a radius of 61 cm (24 inches) around the trunk of the tree using an EZ-Dose sprayer operating at a pressure of 3.1 bar (45 psi) and flow rate of 14 L (3.7 gallon) per minute. Granular applications of Aldicarb were made by placing a weighed amount of product within two, 0.91 m (3 ft) furrows approximately 0.61 m (2 ft) from the base of the two opposing sides of tree. Furrows were covered with soil after application. NUQ 05054 is a slow release imidacloprid and was applied in a 1.22 m (4 ft) circle around the base of the tree.

### Treatment evaluation

ACP was sampled on 3 to 5 central trees in each sprayed plot (10 to 12 in soil drench plots). Adults were monitored by counting those falling on a clipboard covered with a 22×28 cm (8½×11 inch) laminated white sheet held horizontally under a randomly chosen branch which was then struck sharply three times with a PVC pipe to make a count for one “tap” sample [9], [33]. Four tap samples were conducted per tree. Adults on small trees in drench trials were counted visually. Ten randomly selected shoots per plot were collected and examined under a stereoscopic microscope in the laboratory to count number of ACP nymphs per shoot. Observations on adults and nymphs continued until significant differences with the untreated control were observed.

### Statistical analysis

Each test was conducted using 4 replicates for treatment and control so that the probability of detecting treatment effects could be evaluated with confidence. Data were subjected to analysis of variance (ANOVA) to evaluate treatment effects on ACP and means separated using Least Significant Difference (LSD) test contingent on a significant *F* for treatment effect (P = 0.05) [32]. Active ingredients were ranked by the number of days significantly fewer ACP nymphs or adults were observed on treated trees compared to untreated controls (P<0.05). Means averaged over all trials for each active ingredient are presented with standard error.

## Results

### Effects of foliar sprays of insecticides on *Diaphorina citri* adults

Thirty eight of the 42 products tested significantly suppressed adults (*P*<0.05) compared to untreated control (Table 2). A mean reduction of 90–100% in adult psyllids over 24–57 days (3–8 weeks) compared to untreated control was observed with foliar sprays of tolfenpyrad (Apta 15 SC), chlorantraniliprole + thiamethoxam (VoliamFlexi), lamda-cyhalothrin (Warrior II), methidathion (Supracide 2 E), fenpropathrin (Danitol 2.4 EC), abamectin + thiamethoxam (Agri-Flex), dimethoate (Dimethoate 4 E), chlorpyrifos (Lorsban 4 E) and chlorpyrifos + zeta-cypermethrin (Stallion). Reduction with the high rates of the above products and flupyradifurone (Sivanto 200 SL), thiamethoxam (Actara 25 WG), fenpyroximate (Portal), naled (Dibrom 8 E), sulfoxaflor (Closer SC), spinetoram (Delegate WG), zeta-cypermethrin (Mustang Max 1.5 EC), fenazaquin (Magus), spirotetramat (Movento 240 SC), diflubenzuron (Micromite 80 WGS), *Chromobacterium subtsugae* (Grandevo), phosmet (Imidan 70 W), *Burkholderia* spp (MBI-206 EP), potassium salts of fatty acids (M-Pede) and pyridaben (Nexter) also averaged 90% or more.

Reduction averaged 38–88% over 16–67 day (2–9 week, Table 2) periods with high rates of chlorantraniliprole (Exirel), beta-cyfluthrin (Baythroid XL), abamectin (Agri-Mek 0.15 EC), potassium silicate (Sil-Matrix), imidacloprid (Admire Pro), 435 oil, flubendiamide (Belt 4 SC), pymetrozine (Fulfill 50WDG), spinosad (Entrust SC), oxydemeton-methyl (MSR 2 E), carbaryl (Sevin XLR), *Isaria fumosoroseus* (NoFly WP) and *Chenopodium ambrosioides* (Requiem 25 EC). Psyllid reduction associated with low rates was much less compared with high rates and never reached 90%. We did not observe apparent suppression of adults from acetamiprid (Assail 30 SG), azadirachtin (Aza-direct), spirodiclofen (Envidor 2 SC) or pyrifluquinazon (NNI0101-20SC).

Application of some insecticides with HMO improved their performance against ACP (Table 4). Significant suppression of adults prolonged 7–14 days and improved 9–47% with the addition of an adjuvant to treatments of fenpropathrin (Danitol 2.4 EC), sulfoxaflor (Closer SC), spinetoram (Delegate WG) and diflubenzuron (Micromite 80 WGS). HMO by itself provided an average of 36% reduction in adults for 18 days (Table 2).

### Effects of foliar sprays of insecticides on *Diaphorina citri* nymphs

Forty of the 42 products provided some level of significant nymphal suppression (*P*<0.05, Table 3). More than 90% of mean reduction lasting 17–24 days was observed with tolfenpyrad (Apta 15 SC), chlorantraniliprole + thiamethoxam (VoliamFlexi), abamectin + thiamethoxam (Agri-Flex), lamda-cyhalothrin (Warrior II), spinetoram (Delegate WG), and dimethoate (Dimethoate 4 E). Similar levels of reduction were observed with high rates of these six products and phosmet (Imidan 70 W), sulfoxaflor (Closer SC), chlorpyrifos (Lorsban 4 E), zeta-cypermethrin (Mustang Max 1.5 EC), spirotetramat (Movento 240 SC), fenpropathrin (Danitol 2.4 EC), fenazaquin (Magus), flupyradifurone (Sivanto 200 SL), thiamethoxam (Actara 25 WG), *Chromobacterium subtsugae* (Grandevo), fenpyroximate (Portal), potassium salts of fatty acids (M-Pede), imidacloprid (Admire Pro), oxydemeton-methyl (MSR 2 E) and carabryl (Sevin XLR). From remaining 21 products 19 provided 40–89% reduction in nymphs at high rates. Psyllid reduction associated with low rates was much less compared with high rates and never reached 90% except for chlorantraniliprole + thiamethoxam (VoliamFlexi) and lamda-cyhalothrin (Warrior II). Only azadirachtin (Aza-direct) and pyrifluquinazon (NNI0101-20SC) showed no activity against nymphs as with adults. Nymphal suppression prolonged 3–17 days and improved 1–78% by adding HMO to insecticidal sprays except fenpyroximate (Portal) (Table 4). HMO by itself provided an average of 50% reduction in nymphs for 9 days (Table 3).

### Effects of soil applications of insecticides on *Diaphorina citri* adults and nymphs

Mean reduction (*P*<0.05) of 78–85% of adults over 81–111 days (12–16 weeks) and 71–89% of nymphs over 85–107 days (12–15 weeks) was observed with soil drenches of imidacloprid (Admire Pro 4.6 SC), thiamethoxam (Platinum 75 SG) and clothianidin (Belay 2.13 SC) applied to young trees (Table 5). Comparable reduction of both adults and nymphs was observed with the soil drenches of cyantraniliprole (Verimark). Up to 100% reduction lasting 245–296 days in nymphs was observed with Verimark, Admire Pro 4.6 SC and Platinum 75 SG. A slow release granular formulation of imidacloprid (NUQ 05054) lasted longer than liquid formulations. Aldicarb (Temik 15 G) was less effective than imidacloprid and cyantraniliprole but provided greater suppression compared to dinotefuran (Venom 70 SG), flupyradifurone (Sivanto 200 SL) and spirotetramat (Movento MPC), both in duration and magnitude. Flonicamid (Beleaf 50 SG) was the least effective insecticide tested as a drench although it also showed activity. Psyllid reduction associated with low rates was much less compared with high rates.

## Discussion

Insecticides are the most important component of ACP management available to reduce the spread and severity of HLB. Therefore, repeated field evaluations of multiple products against ACP are needed to provide growers with a range of effective products with different MoA that can be rotated to suppress ACP and delay the evolution of insecticide resistance. We observed that sprays of 23 products including new additions tolfenpyrad, cyantraniliprole, flupyradifurone and sulfoxaflor representing 9 known IRAC MoAs and 3 unknown MoAs provided more than 90% reduction in psyllid populations. Tolfenpyrad, cyantraniliprole and flupyradifurone provided more ACP reduction than sulfoxaflor and were comparable to previously registered insecticides both in duration and magnitude of psyllid reduction. Tolfenpyrad, cyantraniliprole and sulfoxaflor are now registered for use against ACP in the USA. Cyantraniliprole is a second-generation anthranilic diamide insecticide MoA group 28 responsible for activating ryanodine receptors and negatively impacting muscle functions. Significant reduction in the ACP with cyantraniliprole compared to a commonly used pyrethroid fenpropathrin (Danitol 2.4 EC) was also observed in the laboratory and field in another study [76]. Tolfenpyrad is classified as MoA group 21A as is fenazaquin, fenpyroximate and pyridaben. Sulfoxaflor and flupyradifurone belong to MoA groups 4C and 4D respectively, thus different sub groups than other (4A) neonicontinoids. Flupyradifurone (Sivanto) is from the butenolide chemical class, containing a bioactive scaffold originally isolated from the plant *Stemona japonica*. Premixes Agri-Flex (abamectin 3% + thiamethoxam 13.9%) and VoliamFlexi (chlorantraniliprole 20% + thiamethoxam 20%) showed comparable effectiveness, but with the disadvantage of removing two modes of action from rotation. These new and promising insecticides along with several already registered products which showed high levels of effectiveness against ACP will broaden the range of products available to control this pest. Others that have shown activity against sucking pests and citrus leafminer may also prove effective [35], [38], [77], [78].

Although the number of times a product was tested varied from 1 to 24, each test was conducted rigorously using 4 replicates for treatment and control so that the probability of detecting treatment effects could be evaluated with confidence. Obviously, more tests would warrant even greater confidence in the result. Generally, findings on effective products were similar when tested multiple times, and in some cases were also confirmed in studies by others. Application of insecticides with HMO as adjuvant generally improved their effect against ACP. The petroleum based HMO, itself formulated with a surfactant, is a commonly used adjuvant which, when applied alone, also provided considerable protection from ACP (Table 2,3).

Insecticides approved by Organic Management Research Institute (OMRI) such as petroleum based HMO (435 oil), potassium salts of fatty acids (M-Pede), potassium silicate (Sil-Matrix) (mineral product), spinosad (Entrust SC) and *Chromobacterium subtsugae* (Grandevo) (bacterial cultures or extracts) provided an average of only two weeks of control. However, 74–97% suppression was seen at high rate which is comparable to standard synthetic insecticides. While the effectiveness of these products tended to be short-lived, they could still be useful for rotation with synthetic insecticides to reduce selection for insecticide resistance in psyllids, conserve natural enemies, for application on blooming citrus for which few synthetic products are allowed, and in organic groves that prohibit synthetic products [45], [79]. Frequent applications of such insecticides during the growing season may be an option compatible with biological control.

Most foliar sprays appeared to suppress adults longer than nymphs with the exceptions of acetamiprid (Assail 30 SG), spirodiclofen (Envidor 2 SC), phosmet (Imidan 70 W) and pyridaben (Nexter). This may largely be due to the short (3-week) duration of shoot suitability for oviposition and subsequent nymphal development, after which there is little or no new flush that has been sprayed. Thus, direct effects on nymphs are measurable for only the 2–3 weeks it takes for new shoots to harden and for nymphs to mature to adulthood. Later effects on adults and subsequently nymphal populations are probably carryovers from earlier suppression.

Several products such as 435 oil, imidacloprid (Admire Pro), acetamiprid (Assail 30 SG), flubendiamide (Belt 4 SC), sulfoxaflor (Closer SC), spirodiclofen (Envidor 2 SC), pymetrozine (Fulfill 50WDG), phosmet (Imidan 70 W), spirotetramat (Movento 240 SC), oxydemeton-methyl (MSR 2 E) and flupyradifurone (Sivanto 200 SL) provided 13–43% more reduction of nymphs than adults. However, most young nymphs are protected inside the newly developing unfolded leaves where ACP oviposit, therefore some avoid contact with the spray. These residual populations along with some left over adults are usually enough to initiate a new generation that may require additional treatment.

*Diaphorina citri* populations respond rapidly to selection pressures due to high fecundity and short generation times, so any insecticide application selects for resistance. Some degree of resistance to key insecticides has already been documented in ACP populations in Florida [76]. Therefore, it is prudent to use a particular MoA only once a year. There is no “fits all” spray program that will satisfy every grower's needs in regard to cost, efficacy against ACP and other pests, conservation of beneficial insects and resistance management. Example programs based on number of sprays per year using currently registered products are given in Table 6 to illustrate how these criteria could be used, contingent on actual pest populations and individual or regional assessment of all factors.

**Table 6:** Example insecticide spray programs for *Diaphorina citri* considering other pests in Florida citrus.

	Insecticide sprays per year (excluding frequent applications of 435 oil alone)						
Month	One	Two	Four	Five	Seven	Other pests controlled	MOA^c^ Group
Jan	Pyrethroid	Pyrethroid	Pyrethroid	Pyrethroid	Pyrethroid	weevils	3
Feb			Movento^a^ ^,b^	Movento^a^ ^,b^	Movento^a^ ^,b^	rust mites, scales, mealybugs	23
Mar					Portal^b^	spider mites, rust mites	21A
					Closer	aphids, mealybugs	4C
Apr	435 Oil	435 Oil	435 Oil	435 Oil	435 Oil	leafminer, rust mites	NA
May	435 Oil	435 Oil	Delegate^a^	Delegate^a^	Delegate^a^	leafminer	5
Jun			Delegate^a^	Delegate^a^	Agri-Mek^a^ ^,b^ or Agri-Flex^a^ ^,b^	leafminer, rust mites	6 or 4+6
Jul	435 Oil	435 Oil	435 Oil	435 Oil	435 Oil	leaf miner, rust mites	NA
Aug							
Sep				Micromite^a^ ^,b^	Micromite^a^ ^,b^	leafminer, rust mites, weevils	15
Oct							
Nov-Dec		Organophosphate	Organophosphate	Organophosphate	Organophosphate	weevils	1B

a Generally applied with oil or another surfactant. ^b^ Primarily for control of nymphs.

c Insecticide Resistance Action Committee (IRAC) Mode of Action (MOA) group, http://www.irac-online.org, NA  =  Not available.

Young trees flush often and are best protected with soil drenches or possibly injections of systemic insecticides. In the past, the neonicotinoids imidacloprid, thiamethoxam, and clothianidin were the only effective systemic insecticides allowed in Florida citrus for drench application, providing extended protection against ACP and, to a lesser extent, citrus leafminer. However, all shared the same 4A MoA and therefore could only be rotated with sprays of different MoA to slow selection for pesticide resistance. Cyantraniliprole (MoA 28) has been shown to provide significant reduction of ACP as well as citrus leafminer as a drench comparable to the neonicotinoid insecticides [64], [65] and can thus serve as a rotation partner to slow the evolution of insecticide resistance in ACP and other pests.

Growers and managers often work with annual budgets based on anticipated needs and profits. Nevertheless, some flexibility is desirable to account for changes in the actual disease, pest or price situation. Research has shown that 4–7 sprays targeting ACP in citrus orchards with close to 100% HLB incidence significantly increased yields, but were not always cost effective when combined with foliar nutrient sprays to mitigate effects of HLB [80]. Slowing spread of HLB under low incidence conditions would have more far reaching implications but has not be quantitatively evaluated in the US, although aggressive vector control coupled with rogueing of symptomatic trees is purported to be successful on large citrus plantations in Brazil [81]. A further consideration is the effect of resident ACP populations on the sustainability of new citrus plantings in the face of HLB for which economic analysis is not yet forthcoming.

At least one and preferably two aerial or ground applications of broad-spectrum insecticides during the “dormant” (winter) season when most mature trees are not flushing has been shown to provide significant reduction in ACP and need for insecticides into growing season as well as conserving biological control [22], [23], [82]. However, timing of insecticide application, choice of products, and application methods during the growing season are far from standard, given the plethora of factors mentioned above. Several commonly use insecticides were shown to negatively impact predators and parasitoids of ACP in the laboratory and field experiments [33], [83], [84]. Effective use of selective insecticides such as soil-applied systemic insecticides, horticultural mineral oils, lipid synthesis inhibitors and spinosyns can be integrated with sprays of less selective chemistries to reduce ACP populations, risk of pest resistance to insecticides and incidence of HLB. Additional sprays during the growing season could be based on scouting and targeted at adults prior to anticipated new growth to ensure that new growth is protected from infestation.

Our intention here is to furnish a starting point for planning a management program for ACP and other citrus pests. Efficacy is only one, albeit an important consideration in the decision of what insecticide to apply. Grower experience and additional field testing will provide more information on these and new products not presently available. Applications made to larger areas of commercial citrus using an area-wide approach appear to have provided extended suppression by avoiding re-colonization of treated groves from surrounding untreated habitats (www.flchma.org), although other factors of scale, environmental or biological conditions and insecticide resistance may influence outcomes.

Some low levels of resistance against imidacloprid, chlorpyriphos, thiamethoxam, malathion and fenpropathrin have been reported [76]. It is possible that there were some resistant populations at the study locations. The high level of suppression (90-100%) observed over 24-68 days with sprays of 24 insecticides and even longer with soil applied insecticides indicate that resistance levels, if any, were probably low. However, insecticide resistance may become a serious problem for future ACP control, given the increasing intensity of use. Therefore, individual and area-wide management programs need to consider proper pest monitoring and rotation of insecticide MoA. Extensive monitoring for field resistance is also warranted and already initiated in Florida and elsewhere.
